# Probiotics may delay the progression of nonalcoholic fatty liver disease by restoring the gut microbiota structure and improving intestinal endotoxemia

**DOI:** 10.1038/srep45176

**Published:** 2017-03-28

**Authors:** Li Xue, Juntao He, Ning Gao, Xiaolan Lu, Ming Li, Xiaokang Wu, Zeshi Liu, Yaofeng Jin, Jiali Liu, Jiru Xu, Yan Geng

**Affiliations:** 1 Department of Laboratory, The Second Affiliated Hospital of Medical College of Xi’an Jiaotong University, Xi’an, Shaanxi 710004, P.R. China; 2 Department of Gastroenterology, The Second Affiliated Hospital, Xi’an Jiaotong University, Xi’an, Shaanxi 710004, P.R. China; 3 Department of Cardiovascular Surgery, The First Affiliated Hospital of Medical College of Xi’an Jiaotong University, Xi’an, Shaanxi 710061, P.R. China; 4 Department of Pathology, The Second Affiliated Hospital of Medical College of Xi’an Jiaotong University, Xi’an, Shaanxi 710004, P.R. China; 5 Department of Immunology and Pathogenic Biology, Health Science Center, Xi’an Jiaotong University, Xi’an, Shaanxi 710061, P.R. China

## Abstract

Gut-derived bacterial lipopolysaccharide (LPS) and subsequent hepatic toll-like receptor 4 (TLR4) activation have been recognized to be involved in the onset of diet-induced nonalcoholic fatty liver disease (NAFLD), but little is known about the variation of LPS and TLR4 during the progression of NAFLD. Probiotics were able to inhibit proliferation of harmful bacteria and improve gastrointestinal barrier function. However, it’s unclear whether LPS/TLR4 is involved in the protection effect of probiotics on NAFLD. In this study, we described characteristic of gut microbiota structure in the progression of NAFLD, and we also analyzed the relationship between gut microbiota and LPS/TLR4 in this process. Furthermore, we applied probiotics intervention to investigate the effect of probiotics on gut flora structure, intestinal integrity, serum LPS, liver TLR4 and liver pathology. Our results showed that serum LPS and liver TLR4 were highly increased during progression of NAFLD, with gut flora diversity and gut mircobiological colonization resistance (B/E) declining. Furthermore, probiotics could improve gut microbiota structure and liver pathology. Probiotics could also downregulate serum LPS and liver TLR4. Our results suggested that both gut flora alteration and endotoxemia may be involved in the progression of NAFLD. Probiotics may delay the progression of NAFLD via LPS/TLR4 signaling.

Nonalcoholic fatty liver disease (NAFLD), the hepatic manifestation of metabolic syndrome^1^, is characterized by fat accumulation in the liver without significant alcohol consumption^2^. With the increased prevalence of obesity, the number of patients with NAFLD is rapidly increasing over the past 20 years. Currently, NAFLD has become the most common cause of chronic liver disease worldwide^3^,^4^,^5^. NAFLD includes a spectrum of disease ranging from simple steatosis to inflammatory steatohepatitis. Simple steatosis (NAFL), a less severe form of NAFLD, is generally considered to be a benign condition whereas nonalcoholic steatohepatitis (NASH) is a severe form of NAFLD with the prevalence of 2–5% in the general population^6^. The pathological features of NASH include hepatocyte injury, inflammation, and various degrees of fibrosis besides steatosis^7^. NASH is a major risk factor in hepatic cirrhosis, hepatocellular carcinoma and other complications of portal hypertension^8^,^9^,^10^,^11^. Recently although there has been tremendous progress in understanding the pathogenesis of NAFLD, the key factors contributing to the progression of NAFLD from NAFL towards NASH remain incompletely understood.

Gut microbiota have emerged as an important environmental factor involving in the pathogenesis of NAFLD^12^,^13^. The gut microbiota has overall more than 100 fold genes as compared to the host, and is called the “metagenome”^14^. There is an anatomical link between intestine and liver via the hepatic portal system. Based on the connection between intestine and liver, also termed gut-liver axis, gut microbiota and their metabolic by-products may influence liver pathology^15^. The gut barrier is a direct physical barrier against translocation of gut bacteria and bacteria-derived products into the blood. A previous study revealed that dietary fat and glucose could lead to gut barrier injury and increased gut permeability to bacteria and its derived products^16^. The main bacterial bioproduct that is likely to be involved in NAFLD pathogenesis is lipopolysaccharide (LPS), the active component of endotoxin. Gut-derived bacterial LPS is brought to the liver by the portal circulation, thereby activating Kupffer cells, the resident hepatic macrophages by combining with Toll-like receptor 4 (TLR-4) complexes on the cell surface and subsequently inducing the production of inflammatory cytokines^17^,^18^. It was reported that serum LPS and liver TLR-4 were involved in the onset of diet-induced NAFLD animal models^19^. However, little is known about the variation of LPS and TLR-4 during the progression of NAFLD.

Recently, an important role of probiotics in host health has attracted more and more attentions. Li *et al*. first reported that manipulation of the intestinal flora in experimental animals could influence obesity-related fatty liver disease^20^. Probiotics are defined by the Food and Agriculture Organization and World Health Organization (FAO/WHO) as live microorganisms that have a benefit to the host when administered in adequate amounts^21^. Probiotics can include elements of the normal human flora and they can help to maintain the balance of gut microbiota. The most commonly used strains as probiotics are members of *Lactobacilli* and *Bifidobacteria* groups which are included in many functional foods and dietary supplements^22^. Accumulating evidences have shown that probiotics were able to inhibit the proliferation of harmful bacteria and improve gastrointestinal barrier function^23^,^24^,^25^,^26^,^27^. Alteration of intestinal tight-junction protein occludin, is a major molecular mechanism contributing to the increased intestinal permeability. The damage of gut integrity may lead to intestinal endotoxemia and subsequent TLR4 activation in the liver, which is implicated in the pathogenesis of NAFLD^28^. However, it’s unclear whether probiotics could alleviate endotoxemia by modulating gut bacteria and whether endotoxin (LPS)/TLR4 are involved in the protection effect of probiotics on NAFLD.

In this study, we described the characteristic of gut microbiota in the progression of NAFLD, and we also analyzed the relationship between gut microbiota and LPS/TLR4 in this process. Furthermore, we applied probiotics intervention to investigate the effect of probiotics on gut flora disturbance, intestinal integrity, serum LPS, liver TLR4 expression and liver pathology. Our results suggested that both gut flora disorder and endotoxemia may be involved in the pathogenesis and progression of NAFLD. Probiotics could delay the progression of NAFLD. This study assessed the effect of probiotics on NAFLD progression from the view of intestinal microecology and will provide theoretical foundation for targeting gut flora in dietary therapy of NAFLD.

## Results

### Microbiota characteristic in the pathogenesis and progression of NAFLD

**Figure 1:**
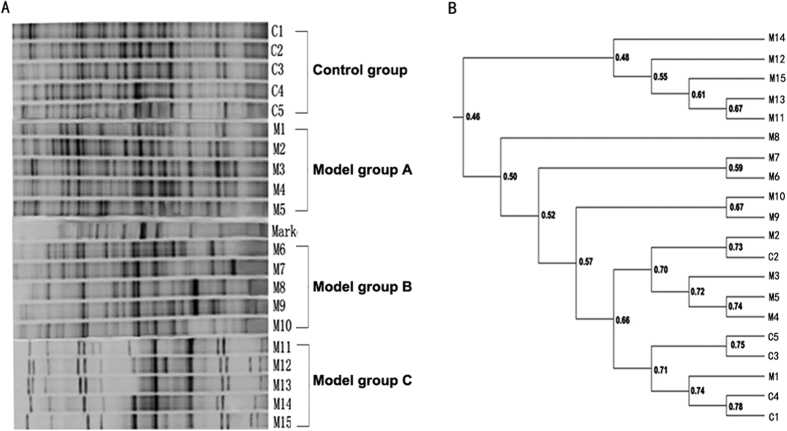
The variability of intestinal bacterial communities as determined by DGGE analysis of samples from the feces. (**A**) Representative DGGE profiles of the fecal samples from control group and model group (A, B and C). C1 to C5 were representative of the control group, M1 to M5 were representative of the model group A, M6 to M10 were representative of the model group B, M11 to M15 were representative of the model group C. (**B**) Dendrogram generated from the bacterial community fingerprints of the feces, the similarities between mucosal specimens were shown in the dendrogram. Dice’s band-matching coefficient and unweighted pair group method with arithmetic averages (UPGMA) were employed to analyze the results.

PCR-DGGE fingerprinting results showed that the diversity of gut flora in model C group were notably less than that of control group and model group A (Fig. 1A). Cluster analysis revealed that similarity coefficient in the same group was higher than that between groups. The similarity coefficient within control group was the highest (69.16% ± 4.64%). The similarity coefficient within model group A, model group B and model group C were 69.48% ± 2.36%, 50.84% ± 3.61% and 56.21% ± 11.06%, respectively. For similarity between groups, the similarity coefficient was 66.61% ± 5.58% between control group and model group A. The similarity coefficient between control group and model group B was 51.22% ± 7.47%. Of note, model group C showed the lowest similarity with control group and the similarity coefficient was just 49.46% ± 4.69% (Fig. 1B).

**Figure 2:**
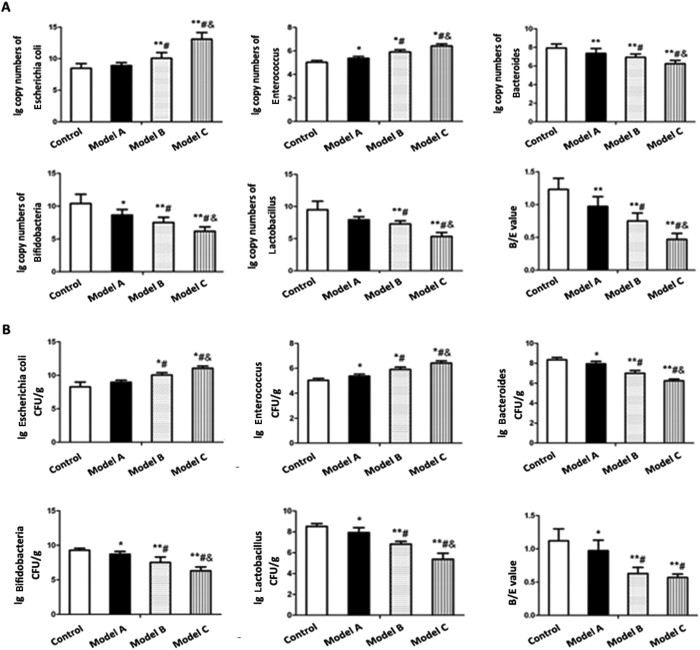
Comparison of five representative bacteria in feces of control group and model group A, B and C. (**A**) Quantification of representative bacteria in feces by quantitative PCR analysis (**B**) Quantification of representative bacteria in feces by traditional culture method. The ratio of *Bifidobacteria* to *Escherichia coli* was used to indicate B/E value representing gut mircobiological colonization resistance. Data represent the mean ± SD of each group. *P < 0.05 compared to the control group; **P < 0.01 compared to the control group; ^#^*P* < 0.05 compared to the model group A; ^&^*P* < 0.05 compared to the model group B.

To furtherly assess alterations in gut microbial composition, we detected *Escherichia coli, Enterococcus, Lactobacillus, Bifidobacteria* and *Bacteroides* in the fecal samples of rats from control group and model group A, B and C using traditional culture method and QT-PCR analysis. As shown in both Fig. 2A and B, amounts of *Escherichia coli* and *Enterococcus* increased with prolonged feeding period. *Escherichia coli* and *Enterococcus* in model group B and C were significantly elevated compared with control group. Significant differences were observed between model group A and control group for *Enterococcus* but not for *Escherichia coli*. Moreover, the amounts of *Escherichia coli* and Enterococcus in both model group B and model group C were significantly increased as compared to model group A, and it was also significantly different between model group B and model group C. For anaerobic bacteria, the levels of *Lactobacillus, Bifidobacteria* and *Bacteroides* in all three model groups were all decreased as compared to control group. Model group B and C had a significant decrease in these anaerobic bacteria relative to model group A, and there were also significant difference between model group B and model group C. Analysis of B/E, employed as an indicator of gut flora colonization resistance, revealed a significant reduction in all three model groups compared with control group. It was observed that B/E value in both model group B and model group C was significantly higher than that in model group A. B/E value was also significantly different between model group B and model group C for RT-PCR analysis but not for traditional culture method.

### Variations of the serum LPS and liver TLR4 expression in NAFLD models

**Figure 3:**
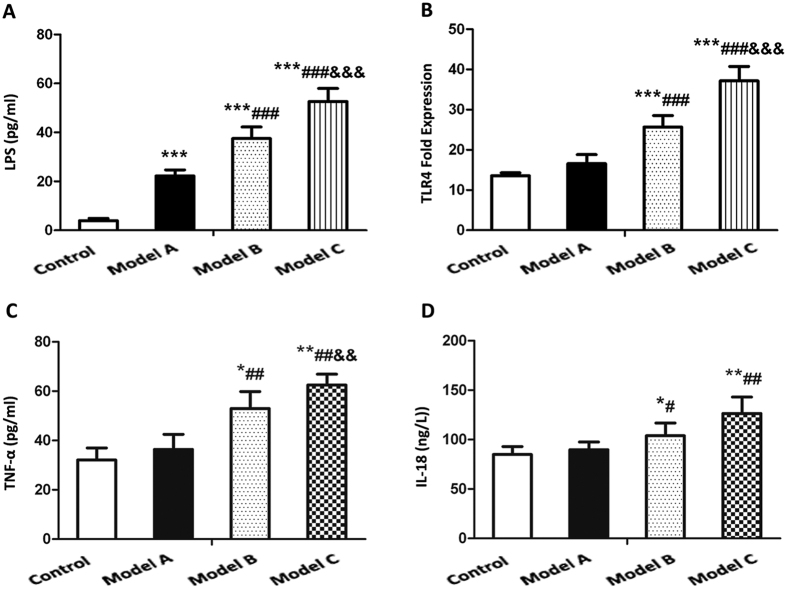
Comparison of serum LPS level, liver TLR4 expression and serum inflammatory cytokines in the control group and model group A, B and C. (**A**) Serum LPS concentration by ELISA (**B**) Liver TLR4 expression by quantitative PCR analysis. Data represent the mean ± SD of each group. *P < 0.05 compared to the control group; **P < 0.01 compared to the control group; ***P < 0.001 compared to the control group; ^#^*P* < 0.05 compared to the model group A; ^##^*P* < 0.01 compared to the model group A; ^###^*P* < 0.001 compared to the model group A. ^&^*P* < 0.05 compared to the model group B; ^&&^*P* < 0.01 compared to the model group B. ^&&&^*P* < 0.001 compared to the model group B.

As shown in Fig. 3A, the concentration of serum LPS showed a rising trend in model group A, B and C as compared to control group. Serum LPS was increased in model group B and C as compared to model group A, and the increased proportion was higher in model group C than that in model group B.

For liver TLR4-mRNA expression, we observed a notably higher levels in model group B and C than that in control group (*P* < 0.001), but there was no difference between model group A and control group (*P* > 0.05). Model group B and C had a significant increase in levels of serum LPS relative to model group A (*P* < 0.05), and there was also significant difference between model group B and model group C (Fig. 3B).

Moreover, there was a positive correlation between serum LPS and the amounts of aerobic bacteria such as *Escherichia coli* and *Enterococcus* while a negative correlation was observed between serum LPS and amounts of anaerobic bacteria such as *Lactobacillus, Bifidobacteria* and *Bacteroides*. For the correlation between liver TLR4 and gut bacteria, there was a similar trend. Notably, we found a negative correlation between serum LPS and intestinal flora B/E value (r_culture_ = −0.709 and r_PCR_ = −0.797). The expression of liver TLR4-mRNA was also negatively associated with B/E value (r_culture_ = −0.723 and r_PCR_ = −0.822) (Table 1).

### Variations of the serum inflammatory cytokines in NAFLD models

As shown in Fig. 3C and D, TNF-α and IL-18 were also increased in model group B and C compared to that in control group (*P* < 0.05), while no significant difference was found between model group A and control group. Both model group B and model group C exhibited a rising trend in serum levels of TNF-α and IL-18 compared with model group A. The increased proportion of IL-18 and TNF-α was significantly higher in model group C than that in model group B (*P* < 0.01). For IL-18, no significant increase was found in model group C as compared to model group B.

### Probiotics reduce body weight in NAFLD models

**Figure 4:**
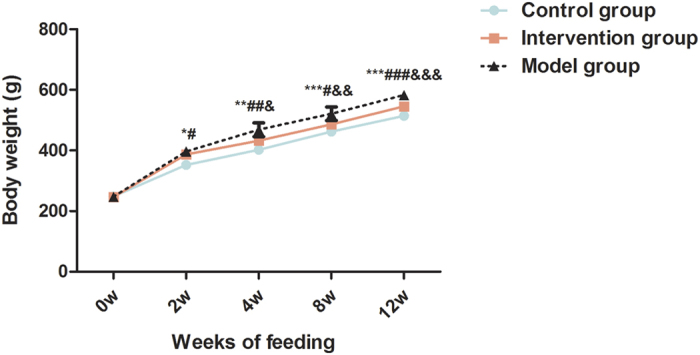
Effects of probiotics on body weight in NAFLD models. Data represent the mean ± SD of each group. *P < 0.05 model group versus control group; **P < 0.01 model group versus control group; ***P < 0.001 model group versus control group; ^#^*P* < 0.05 intervention group versus control group; ^##^*P* < 0.01 intervention group versus control group; ^###^*P* < 0.001 intervention group versus control group; ^&^*P* < 0.05 intervention group versus model group; ^&&^*P* < 0.01 intervention group versus model group; ^&&&^*P* < 0.001 intervention group versus model group.

As shown in Fig. 4, after feeding for 2 w, rats in both model group and intervention group gained significantly more weight than that in the control group. Notably, rats in model group gained significantly more weight than that in the intervention group after feeding for 4 w, and the increased proportion was higher with the prolonged feeding period.

### Probiotics ameliorate gut microbiota dysbiosis in NAFLD models

**Figure 5:**
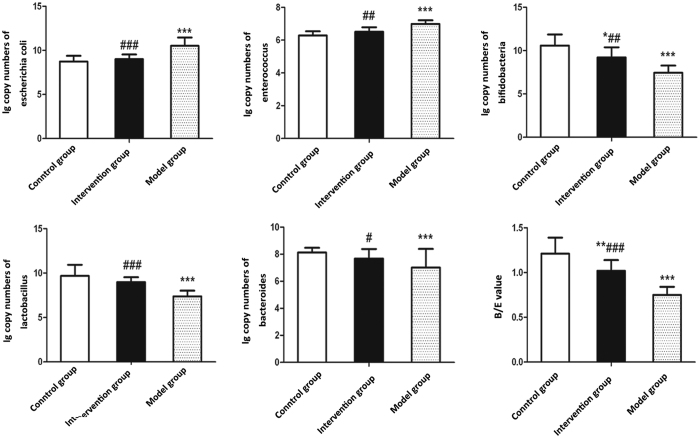
Effects of probiotics on gut microbiota dysbiosis in NAFLD models. Quantitative PCR analysis of fecal five representative bacteria. Data represent the mean ± SD of each group. *P < 0.05 compared to the control group; **P < 0.01 compared to the control group; ***P < 0.001 compared to the control group; ^#^*P* < 0.05 compared to the model group; ^##^*P* < 0.01 compared to the model group; ^###^*P* < 0.001 compared to the model group.

As shown in Fig. 5, amounts of both *Escherichia coli* and *Enterococcus* were significantly increased in the model group compared with control group (*P* < 0.01). Oppositely, amounts of anaerobic bacteria including *Lactobacillus, Bifidobacteria* and *Bacteroides* were significantly decreased in the model group compared with control group (*P* < 0.01). Notably, the intervention group showed a rising trend in these anaerobic bacteria but a declining trend in *Escherichia coli* and *Enterococcus* compared to the model group (*P* < 0.05). Consistent with anaerobic bacteria, B/E in the intervention group was obviously higher than that in model group. Although B/E presented a decreasing trend in the intervention group relative to the control group, no significant differences were observed.

### Probiotics ameliorate loss of intestinal barrier integrity in NAFLD models

**Figure 6:**
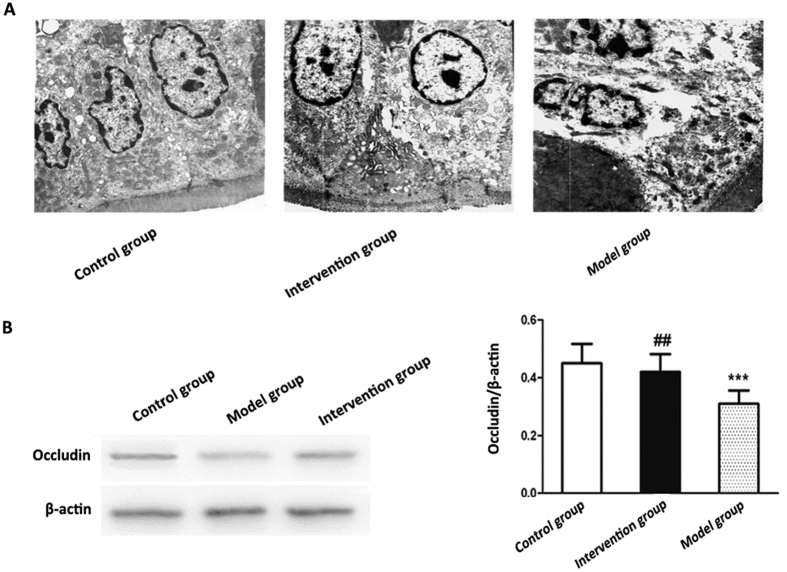
Effects of probiotics on intestinal barrier integrity in NAFLD models. (**A**) Representative images of tight junctions in the jejunal mucosa (transmission electron microscopy, x5000) (**B**) The expression of occludin protein in the intestinal mucosa was detected with β-actin as a loading control by western blotting. Data represent the mean ± SD of each group. *P < 0.05 compared to the control group; **P < 0.01 compared to the control group; ***P < 0.001 compared to the control group; ^#^*P* < 0.05 compared to the model group; ^##^*P* < 0.01 compared to the model group; ^###^*P* < 0.001 compared to the model group.

We examined the tight junctions in the jejunum under a transmission electronmicroscope (TEM) to evaluate the jejunum microstructure. As shown in Fig. 6A, the jejunum in the control group had intact tight junctions and much more regularly aligned and extensive microvilli. However, widened tight junctions and irregularly arranged microvilli were observed in the model group. Of note, tight junctions were more complete and microvilli were more extensive in the invention group than those in the model group. Western-blotting revealed that the level of occludin protein expression was the highest in the intestinal mucosa of control group while the model group had the lowest occludin expression. Moreover, the intervention group displayed a higher expression of occludin than the model group (Fig. 6B).

### Probiotics ameliorate high expression of serum LPS and liver TLR4-mRNA in NAFLD models

**Figure 7:**
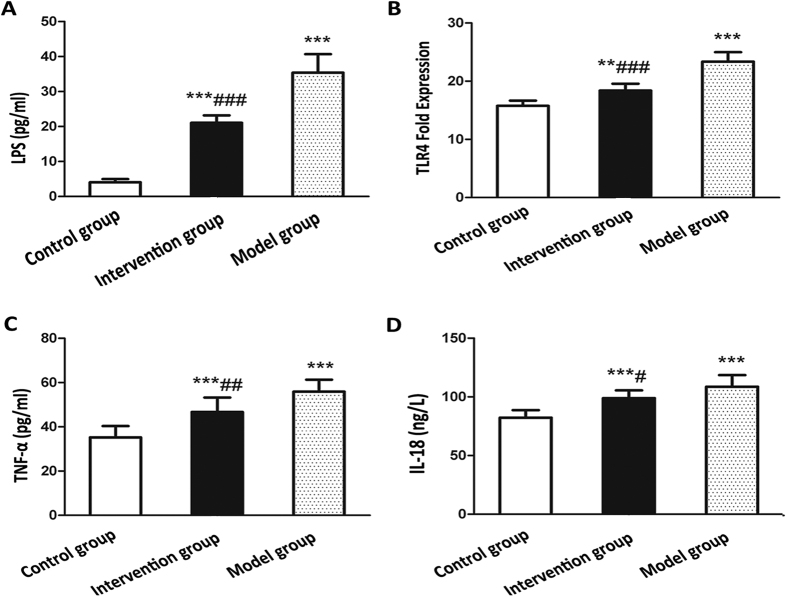
Effects of probiotics on the levels of serum LPS, liver TLR4-mRNA and serum inflammatory cytokines in NAFLD models. (**A**) Serum LPS concentration (**B**) Liver TLR4-mRNA expression (**C**) Serum IL-18 level (**D**) Serum TNF-α level. Data represent the mean ± SD of each group. **P < 0.01 compared to the control group; ***P < 0.001 compared to the control group; ^#^*P* < 0.05 compared to the model group; ^##^*P* < 0.01 compared to the model group; ^###^*P* < 0.001 compared to the model group.

From Fig. 7A and B, it was found that the levels of serum LPS and liver TLR4-mRNA were significantly increased in the model group and intervention group as compared to the control group (*P* < 0.05). Moreover, the intervention group had a lower levels of serum LPS and liver TLR4-mRNA than the model group (*P* < 0.05).

### Probiotics ameliorate serum levels of inflammatory cytokines

As show in Fig. 7C and D, the serum levels of TNF-α and IL-18 were significantly higher in the model group compared with control group and there were significant differences between the two groups (*P* < 0.01). Notably, the serum levels of TNF-α and IL-18 showed an decreasing trend in the intervention group relative to the model group, and significant differences were observed (*P* < 0.05).

### Probiotics ameliorate liver pathology in NAFLD models

**Figure 8:**
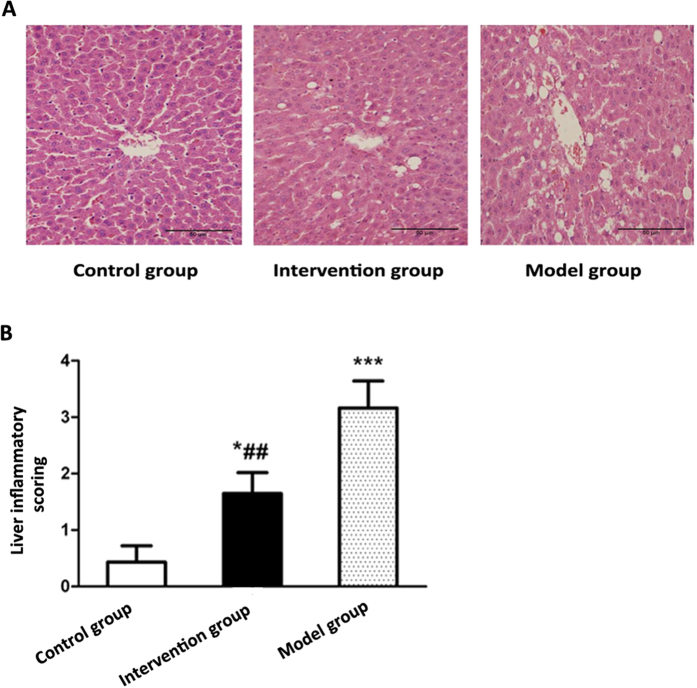
Effects of probiotics on liver pathology in NAFLD models. (**A**) Typical images of representative liver pathology for HE staining (**B**) Statistic analysis of liver inflammation scoring. Data represent the mean ± SD of each group. *P < 0.05 compared to the control group; ***P < 0.001 compared to the control group; ^##^*P* < 0.01 compared to the model group.

Liver histology exhibited that the rats in control group have normal liver histology. In the model group, hepatocyte swelling, disorderly arrangement and inflammatory cells infiltration with large fat droplets emerging were observed. Comparatively, there was just mild steatosis and few infiltrations of inflammatory cells in the intervention group (Fig. 8A). Statistical analysis showed that the degree of liver inflammatory activity was significantly higher in model group and intervention group compared with control group (*P* < 0.01), and it was also significantly different between model group and intervention group (*P* < 0.01) (Fig. 8B).

### Probiotics ameliorate serum levels of liver enzymes and metabolic indices

**Figure 9:**
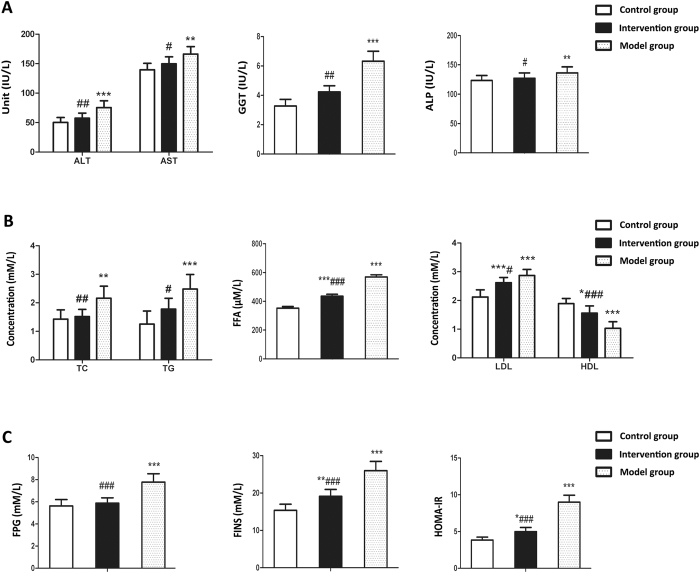
Effects of probiotics on serum levels of liver enzymes and metabolic indices in NAFLD models. (**A**) Serum levels of alanine aminotransferase (ALT), aspartate aminotransferase (AST), gamma-glutamyltransferase (GGT) and alkaline phosphatase (ALP) (**B**) Serum levels of triglyceride (TG), total cholesterol (TC), low-density lipoprotein (LDL), high density lipoprotein (HDL) and free fatty acid (FFA) (**C**) fasting plasma glucose (FPG), fasting insulin (FINS) and homoeostasis model assessment of insulin resistance (HOMA-IR). Data represent the mean ± SD of each group. *P < 0.05 compared to the control group; **P < 0.01 compared to the control group; ***P < 0.001 compared to the control group; ^#^*P* < 0.05 compared to the model group; ^##^*P* < 0.01 compared to the model group; ^###^*P* < 0.001 compared to the model group.

As shown in Fig. 9A, the activity of serum liver enzymes including alanine aminotransferase (ALT), aspartate aminotransferase (AST), gamma-glutamyltransferase (GGT) and alkaline phosphatase (ALP) were significantly higher in the model group compared with control group (*P* < 0.01). The activity of these serum enzymes presented an increasing trend in the intervention group relative to the control group, however, no significant differences were observed. Notably, the activity of these enzymes in the intervention group was decreased compared to that in model group, and significant differences were observed (*P* < 0.05).

For serum lipid, we observed that the model group had higher serum levels of free fatty acid (FFA), triglyceride (TG), total cholesterol (TC) and low-density lipoprotein (LDL) whereas a lower level of high density lipoprotein (HDL) relative to the control group (*P* < 0.05). Importantly, serum levels of TC, TG, LDL and FFA were significantly decreased while that of HDL was remarkably increased in the intervention group as compared to model group (Fig. 9B).

For glycometabolism indices, it was observed that fasting plasma glucose (FPG), fasting insulin (FINS) and homoeostasis model assessment of insulin resistance (HOMA-IR) were significantly higher in the model group compared with control group (*P* < 0.01). Of note, the intervention group had reduced FPG, FINS and HOMA-IR compared with model group, and significant differences were observed (*P* < 0.05) (Fig. 9C).

## Discussion

In this study, genomic DNAs from rats’ fecal samples were amplified with universal primers of V3 region of bacterial 16SrRNA, and then DGGE finger printing techniques were employed to detect the gut microflora diversities and abundances and to analyze the characteristics of gut microbiota structure in the progression of NAFLD. DGGE results showed that control group had a rich diversity in gut microflora. The diversity and abundance of gut microflora in three model groups were persistently decreased as HSHF-feeding period prolonged. These results suggested that the diversity and abundance of gut microflora were closely associated with progression of NAFLD. We also used traditional culture method and QT-PCR to detect the variation of five representative bacteria and gut mircobiological colonization resistance B/E value in the progression of NAFLD. We observed a remarkable increase in aerobic bacteria such as *Escherichia coli* and *Enterococcus* as well as notable decrease in anaerobic bacteria including *Lactobacillus, Bifidobacteria* and *Bacteroides* during the progression of NAFLD. These results demonstrated that there were significant variation of representative bacteria in the gut microflora and decline of gut mircobiological colonization resistance during the progression of NAFLD.

Previous studies demonstrated that the waterfall effect resulting from inflammatory cytokines induced by endotoxin played a pivotal role in the liver injury of NAFLD^29^,^30^. Endotoxin could also directly damage hepatocytes and activate kupffer cells to produce inflammatory cytokines followed by the waterfall effect and release of oxygen radicals, which may cause liver damage. Studies from Giorgio *et al*. demonstrated that the increase of intestinal permeability could promote the elevation of serum LPS^31^ and that the significant increase in serum inflammatory cytokines and intestinal permeability were associated with liver injury of NAFLD^32^. It was also reported that the degree of liver injury was significantly reduced when the NASH model mice were deficient in TLR-4 gene while high expression of TLR4 promoted the liver injury of NAFLD^33^,^34^,^35^. The diet-induced alterations in the hepatic innate immune system have been demonstrated to contribute to obesity-related liver disease^36^. Our results revealed that serum LPS, liver TLR4 expression and serum cytokines in HSHF groups exhibited a rising trend with prolonged HSHF-diet feeding period. We also found that serum LPS, cytokine levels and liver TLR4 expression were negatively associated with gut micro flora B/E value, and serum LPS levels were positively related to liver TLR4 expression. All these results suggested that LPS and TLR4 were key molecules in the pathogenesis of NAFLD, and LPS-TLR4 signaling mediated liver injury may be involved in the progression of NAFLD. Our findings were consistent with other studies demonstrating that there were high levels of serum LPS and liver TLR4 mRNA in NAFLD patients^37^. Recently it was reported that the related inflammatory cytokines could aggravate liver injury and promote the progression of NAFLD from NAFL to NASH^38^,^39^. Our results revealed that with the deepening degree of liver injury, the levels of serum IL-18 significantly increased. These results indicated that IL-18 may be involved in the development of NAFLD. It has been demonstrated that IL-18 not only participated in the inflammation, but also was involved in the regulation of energy metabolism. For example, IL-18 could activate the chemotactic responses of neutrophils and then caused infiltration of inflammatory cells^40^,^41^. In addition, IL-18 could also activate JNK-1 signaling pathway in the adipose tissues which subsequently caused liver steatosis and inhibited the transduction of insulin signaling^42^. Our results also showed that as another inflammatory cytokine, serum TNF-α level was markedly elevated in the model groups compared with control group. The effect of TNF-α may be related to direct cytotoxicity and indirect action that is provoking the release of other inflammatory mediators. Increased TNF-α secretion could activate Kupffer cells of livers to release proinflammatory cytokines and promote hepatocytes apoptosis^43^. TNF-α could reduce insulin sensitivity to its receptor and then be implicated in the process of insulin resistance, ultimately exacerbating liver steatosis^43^. TNF-α could also be involved in the second hit process of NAFLD as TNF-α may intensify the dysfunction of mitochondria and induce the release of cytochrome C from mitochondria, which would increase ROS production and formation of lipid peroxidation and eventually caused liver injury^44^. All these suggested that TNF-α was an important cytokine in the progression of NAFLD from NAFL to NASH^45^.

Recently more and more attention has been paid to the action of gut microecology. Regulating gut flora with probiotics or prebiotics has become a new approach to prevent and treat several metabolic diseases such as NAFLD^46^. Studies from Li *et al*. have suggested that manipulation of the intestinal flora may be novel therapies for NAFLD^20^. In this study, we found that probiotics intervention could inhibit the proliferation of aerobic bacteria while could also upregulate the proportion of anaerobic bacteria and gut flora colonization resistance. These results suggested that probiotics may improve the balance of gut flora. Our findings were consistent with studies demonstrating that probiotics could improve the structure and proportion of micro flora in the gut and then could help to improve the liver injury of NAFLD^47^. LPS, a key molecule emerged from gut microflora, played an important role in the modulation on the early stage of inflammation and metabolic diseases. LPS could upregulate the expression of TLR4 and induce the activation of inflammatory cytokines followed by the waterfall effect, which may accelerate liver injury^48^. Our results showed that serum LPS, liver TLR4-mRNA and serum inflammatory cytokines in probiotics intervention group were all significantly decreased compared to that in model group. Additionally, we found that the degree of liver steatosis and inflammatory cell infiltration in the intervention group was also reduced relative to the model group. Based on these results, we speculated that probiotics may delay the process of NAFLD by inhibiting LPS-TLR4 signaling pathway.

Occludin was regarded as one of the most important tight junction-associated structural proteins in the intestines and played a very important role in keeping physical barrier of intestinal mucosa^49^. Studies from Luca Miele *et al*. demonstrated that impairment of tight junction between the enterocytes could provoke increased intestinal permeability^28^, which may trigger translocation of a great deal of bacteria and bacteria endotoxin into the blood circulation. Importantly, endotoxemia could also induce the activation of inflammatory cytokines, which leaded to hypoxia-ischemia in the local intestinal mucosa and then caused further increase of intestinal permeability^16^,^50^. In this study, we found probiotics could upregulate the expression of intestinal tight junction protein occluding and could improve the integrity of gut, suggesting that probiotics could protect intestinal mucosa barrier.

In sum, probiotics could improve intestinal flora disturbance presented in NAFLD and could also restore the microecosystem of gut flora and upregulate the expression of occludin, which may inhibit bacteria or endotoxin into the blood circulation and lead to the decrease of TLR4 expression in the liver. Thereby probiotics may reduce the hepatic and systemic inflammatory response caused by endotoxin and inflammatory cytokines through LPS/TLR4 signaling. Consequently, probiotics could ameliorate liver inflammation and thus delay or prevent NAFLD progression. Our findings revealed that probiotics supplementation could realize the balance of gut flora, suggesting that dietary intervention targeting gut flora would provide a new approach to prevent and treat NAFLD.

## Method

### Animal

Eight-week-old SPF male SD rats, purchased from SLAC Laboratory (SLAC Laboratory Animal, Shanghai, China). After 1 week of free access to a standard diet, 48 rats were randomly divided into four groups: (i) normal diet fed for 12 w (control group) (n = 12); (ii) HSHF diet fed for 8 w (model group A) (n = 12); (iii) HSHF diet fed for 12 w (model group B) (n = 12); and (iv) HSHF diet fed for 16 w (model group C). Standard diet was comprised of 80% carbohydrate, 15% protein and 5% fat while HSHF diet contained 75% standard diet, 18% lard, 5% sucrose, 2% cholesterol and 0.3% bile salt. All the experimental rats took food and drank water as their pleasure. Rats were housed at 22 ± 3 °C and 60 ± 5% relative humidity in a specific pathogen-free facility maintained on a 12-hour light/dark cycle. The physical action, emotional behaviours, consumption of food and water, and defecation of experimental animal were observed daily.

In probiotics intervention studies, eight-week-old SPF male SD rats were acclimated for 1 week and then randomly divided into three groups, namely normal control group, NAFLD model group and probiotics intervention group with 12 rats in each group. Rats of control group and model group received a standard diet and HSHF diet for 12 weeks, respectively. In probiotics intervention group, rats were administered HSHF diet concomitantly supplemented with probiotics (0.5 g/day/each rat) for 12 weeks. Standard diet was comprised of 80% carbohydrate, 15% protein and 5% fat while HSHF diet contained 75% standard diet, 18% lard, 5% sucrose, 2% cholesterol and 0.3% bile salt. Probiotic supplementation consisted of 0.5 × 10^6^ colony-forming units (CFU) live *Bifidobacterium infantis* and *Lactobacillus acidopilus* and 0.5 × 10^5^ CFU live *Bacillus cereus*. The experiments involving animals were approved by the Animal Ethics Committee of Xi’an Jiaotong University. All treatment procedures were performed in accordance with the guidelines of the Institutional Animal Care and Use Committee of Xi’an Jiaotong University.

### Tissue extraction and animal killing

At the end of prescribed feeding period, all rats were overnight fasted and anaesthetized with an i.p. injection of 10% chloral hydrate (100 μL/20 g body weight). Once rats were anaesthetized, blood was collected from the inferior vena cava. The livers and intestines were excised followed by weighing livers immediately. Liver index was calculated using the following formula:


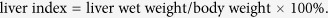


### Histology

Formalin-fixed liver and ileum tissue was processed and 5-μm thick paraffin sections were stained with hematoxylin–eosin (HE) for histological analysis. The images were acquired using Leica microscope system (Leica microsystem, Germany). All sections were examined by the same person who was blinded to treatment status. Five random areas of per tissue section were examined and at least four different sections were examined for each treatment group. Histopathology scoring was evaluated in terms of the degree of liver steatosis, lobular inflammation, ballooning, and liver fibrosis. Liver steatosis was scored as follows: grade 0, no fat or fatty hepatocytes occupying less than 5% of hepatic parenchyma; grade 1, fatty hepatocytes occupying 5–33% of hepatic parenchyma; grade 2, fatty hepatocytes occupying 34–66% of hepatic parenchyma; and grade 3, fatty hepatocytes occupying more than 66% of hepatic parenchyma. The score for lobular inflammation was as follows: grade 0, none; grade 1, focal isolated periportal lymphocytes (<2 foci/field); grade 2, periportal aggregate lymphocytes (2–4 foci/field); and grade 3, intralobular lymphocytes (>4 foci/field). Hepatocellular ballooning was scored as follows: grade 0, none; grade 1, mild ballooning; and grade 2, moderate ballooning; Last, fibrosis was scored as follows: grade 0, absent; grade 1, thin isolated septa; grade 2, periportal fibrosis; and grade 3, periportal and intralobular fibrotic septa.

### Denaturing Gel Gradient Electrophoresis (DGGE)

General bacterial 16S rDNA gene profiles of fecal bacteria in three model groups and control group were generated using the D-Code universal mutation detection system (Bio-Rad, Hercules, CA).Briefly, gels were prepared using 30–70% denaturing gradients containing 8% polyacrylamide. The amplification products of universal primers GC357F and 518 R targeting bacterial 16 S rDNA-V3 region were loaded onto the gel and electrophoresed in 1x Tris-acetate-EDTA buffer (40 mM Tris, 20 mM acetic acid, 1 mM EDTA) (TAE).Gels were run at 60 V for 2.5 h firstly, and then were run at 90 v for 12 h (DGGE-2001, C.B.S. Scientific, San Diego, CA, USA), and stained with SYBRI (Invitrogen, Carlsbad, CA, USA). Gel bands were then visualized with a Typhoon FLA 9500 Molecular Imager (GE Healthcare, Pittsburgh, PA, USA). Community profiles were subjected to cluster analysis using QuantityOne software (version 4.2; BioRad). Dice’s band-matching coefficient and unweighted pair group method with arithmetic averages (UPGMA) were employed to analyze the results.

### DNA and RNA Extraction

DNA extraction of the fecal pellets was carried out by QIAamp stool DNA Mini Kit (QIAGEN, Hilden, Germany) and total RNA was extracted from the liver and then purified using the TRIZOL kit (Invitrogen, Carlsbad, CA, USA) according to the manufacturer’s instructions. The extracted nucleic acid concentration and purity (A260/A280) were measured with a Thermo Scientific Microplate instrument (Thermo Fisher, Waltham, MA, USA). DNA extracts were stored at −80 °C before use in the following procedures. The extract total RNA from liver was immediately performed RT-PCR reaction using a PrimeScript^TM^RT reagent kit (Takara Bio Inc., Shiga, Japan) according to the manufacturer’s instructions.

### RT-PCR

Fecal DNA was subjected to RT-PCR assays to determine the abundance of five targeted bacteria based on the detection of 16S rRNA genes. The primer sequences were as follows: *Escherichia coli* F: (5′-GCCTGATGGAGGGGGATAAC-3′) and R: (5′-TCCTCTCAGACCAGCTAGGG-3′), *Enterococcus* F: (5′-GACGAAAGTCTGACCGAGCA-3′) and R: (5′-TTAGCCGTGGCTTTCTGGTT-3′), *Lactobacillus* F: (5′-AGCAGTAGGGAATCTTCCA-3′) and R: (5′-CACCGCTACACATGGAG-3′), *Bifidobacteria* F: (5′-CTCCTGGAAACGGGTGG-3′) and R: (5′-GGTGTTCTTCCCGATATCTACA-3′), *Bacteroides* F: (5′-GAGGAAGGTCCCCCACATTG-3′) and R: (5′-ACCCATAGGGCAGTCATCCT-3′). The copy number of the target DNA was determined via comparison to the plasmid DNA dilution series standard curves. Bacterial quantity was expressed as log10 copy numbers of bacteria per gram of stool.

Additionally, Liver samples were subjected to RT-PCR to assess mRNA expression of TLR4. The primer sequences were as follows: TLR4-F: (5′-GCCGGAAAGTTATTGTGGTGGT-3′) and TLR4-R: (5′-ATGGGTTTTAGGCGCAGAGTTT-3′), β-actin-F: (5′-CCAAGGCCAACCGCGAGAAGATGAC-3′) and β-actin-R: (5′-AGGGTACATGGTGGTGCCGCCAGAC-3′). The expression of TLR-4 was assessed relative to the housekeeping gene β-actin. The results were normalized to the expression of β-actin gene. The results were quantified using the 2^−ΔΔCT^ method^51^ and expressed as fold change relative to control group. β-actin gene was chosen as the reference gene because of its uniform expression throughout the samples evaluated. The ratio of *Bifidobacteria to Escherichia coli* was used to indicate B/E value representing gut mircobiological colonization resistance.

### Gut bacteria culture

Stool samples of 0.1 g were diluted in series by 10 times to the concentration gradient ranging from 10^1^ to 10^8^, and then cultured in selective culture medium by which *Escherichia coli, Enterococcus, Lactobacillus, Bifidobacteria* and *Bacteroides* were cultured, respectively. Bacteria were grown at 37 °C for 2 days under aerobic conditions or for 3 days under anaerobic conditions. All the isolates were identified using biochemical methods (API 20 A System; BIO Merieux, FR). The bacteria were quantified as logarithm of colony-forming units per gram faeces (log10 CFU/g).

### Assessment for the ultrastructure of intestinal mucosa by transmission electron microscopy

Jejunum mucosa samples were subjected to transmission electron microscopy. The biopsy samples were immediately fixed using 3% glutaraldehyde and kept at 4 °C for 2 hours, then post-fixed with osmium tetroxide and dehydrated with gradientalcohol, and finally the tissues were infiltrated with a 151 solution of Epon 812 and epoxypropane, then embedded in Epon 812 resin. Ultrathin sections were stained with uranyl acetate and lead citrate, and were photographed using a transmission electron microscopy (Philips Tecnai-12 Biotwin, Netherlands). High-magnification (5,000×) images were captured to assess the ultrastructure of the tight junctions in the Jejunum.

### Detection of metabolic indices

Serum levels of liver enzymes including ALT, AST, ALP and GGT, serum lipid including TG, TC, HDL and LDL, and FPG were all determined using an automatic biochemical analyzer. FINS and FFA were measured by enzyme-linked immunosorbent assay (ELISA) according to the manufacturer’s instructions. FINS Quantikine ELISA Kit (e-Bioscience, Inc., San Diego, CA, USA) was used for the quantitative measurement of Insulin. FFA Quantikine ELISA kit was purchased from Zen-Bio (North Carolina, USA). Insulin resistance was evaluated by HOMA-IR. HOMA-IR is the product of fasting blood insulin concentration (mIU⁄L) and glucose concentration (mM⁄L) divided by 22.5.

### Quantification of inflammatory cytokines

ELISA assays were performed to determine serum levels of TNF-α and IL-18. IL-18 and TNF-α ELISA kit were purchased from Life Span (Denver, USA) and R&D (Michigan, USA), respectively. All assays were performed in duplicate, and the absorbance was determined using a microplate reader (Bio-Rad Laboratories, Hercules, CA, USA).

### Western immunoblotting

Proteins were extracted from purified BMMCs using RIPA Buffer (Xianfeng Biotech, Xi’an, China) containing protease and phosphatase inhibitors according to the manufacturer’s instructions. Equal amounts of protein lysates from each group were separated by SDS-PAGE and transferred to a methanol-activated PVDF membrane (Millipore, Beijing, China). The membrane was blocked with Tris-buffered saline containing 0.1% Tween-20 and 5% non-fat milk for 2 h, and then incubated with primary antibody at 4 °C for 24 h, washed, and incubated with secondary goat anti-rabbit antibody conjugated with HRP (Abgent, San Diego, USA). Western blot analysis was conducted according to standard procedures using a Pierce™ ECL Plus Western Blotting Substrate detection kit (Thermo Fisher Scientific, Rockford, USA). Image capture and analysis were performed using a GE Image Scanner (GE Healthcare, USA). Protein levels were normalized to those of β-actin. Primary occludin (ab31721) antibody was from Cell Signaling Technology (Cambridge, MA, USA), and primary β-actin (66009-1) antibody was from Proteintech (Chicago, USA).

### Statistical analysis

Data represent the mean ± SD from at least three independent experiments. All the analysis was performed using SPSS software version 15.0. The results from tests of normality showed that our data were normally distributed. Comparison between groups for continuous data was carried out using one-way analysis of variance (ANOVA) with a Turkey’s multiple comparisons. Comparison between groups for ordinal data was carried out using a Kruskal Wallis test. Correlation analysis was carried out using a Pearson correlation. A *P* value < 0.05 indicated a significant difference.

## Additional Information

**How to cite this article**: Xue, L. *et al*. Probiotics may delay the progression of nonalcoholic fatty liver disease by restoring the gut microbiota structure and improving intestinal endotoxemia. *Sci. Rep.* **7**, 45176; doi: 10.1038/srep45176 (2017).

**Publisher's note:** Springer Nature remains neutral with regard to jurisdictional claims in published maps and institutional affiliations.

## Figures and Tables

**Table 1:** Correlation analysis between serum LPS or liver TLR4-mRNA expression and gut representative bacteria, and between serum LPS or liver TLR4-mRNA expression and B/E value.

	*Escherichia coli*	*Enterococcus*	*Lactobacillus*	*Bifidobacteria*	*Bacteroides*	*B/E*_*C*_	*B/E*_*P*_
LPS	0.675**	0.648*	−0.865***	−0.563*	−0.548*	−0.709**	−0.797**
TLR4	0.688**	0.589*	−0.698**	−0.596*	−0.591*	-0.723**	−0.822***

B/E value representing gut mircobiological colonization resistance was expressed by the ratio of *Bifidobacteria to Escherichia coli*.

B/E_C_: Defined as B/E value when using traditional bacteria culture method.

B/E_P_: Defined as B/E value when using QT-PCR method.

*Defined as *P* < 0.05, **P < 0.01, ***P < 0.001.
